# Distribution and Genetic Diversity of Hepatitis E Virus in Wild and Domestic Rabbits in Australia

**DOI:** 10.3390/pathogens10121637

**Published:** 2021-12-17

**Authors:** Maria Jenckel, Ina Smith, Tegan King, Peter West, Patrick L. Taggart, Tanja Strive, Robyn N. Hall

**Affiliations:** 1 CSIRO Health and Biosecurity, Black Mountain, Canberra, ACT 2601, Australia; Maria.Jenckel@csiro.au (M.J.); Ina.Smith@csiro.au (I.S.); tegan.king4891@gmail.com (T.K.); Tanja.Strive@csiro.au (T.S.); 2 New South Wales Department of Primary Industries, Orange 2800, Australia; Peter.West@dpi.nsw.gov.au; 3 Centre for Invasive Species Solutions, University of Canberra, Bruce, ACT 2601, Australia; Pat.Taggart@dpi.nsw.gov.au; 4 New South Wales Department of Primary Industries, Queanbeyan 2620, Australia

**Keywords:** hepeviridae, rabbits, genomic epidemiology, seroepidemiology, Luminex, serology, whole-genome sequencing

## Abstract

In 2020, Hepatitis E virus (HEV) was detected for the first time in Australian rabbits. To improve our understanding of the genetic diversity and distribution of the virus, 1635 rabbit liver samples from locations across Australia were screened via RT-qPCR for HEV. HEV genomes were amplified and sequenced from 48 positive samples. Furthermore, we tested 380 serum samples from 11 locations across Australia for antibodies against HEV. HEV was detected in rabbits from all states and territories, except the Northern Territory. Seroprevalence varied between locations (from 0% to 22%), demonstrating that HEV is widely distributed in rabbit populations across Australia. Phylogenetic analyses showed that Australian HEV sequences are genetically diverse and that HEV was likely introduced into Australia independently on several occasions. In summary, this study broadens our understanding of the genetic diversity of rabbit HEV globally and shows that the virus is endemic in both domestic and wild rabbit populations in Australia.

## 1. Introduction

Hepatitis E virus (HEV) is a positive sense RNA virus in the genus *Orthohepevirus*. The genome is approximately 7200 bases in length and contains three open reading frames (ORF1, ORF2, ORF3), which encode two polyproteins (ORF1 and ORF2) and a small 113–115 amino acid protein (ORF3) [1]. HEV is genetically diverse and can be classified into 8 genotypes [2]. Up to now, human infections have been reported with genotype 1–4 viruses, along with a single human infection with genotype 7 [3]. While genotype 1 and 2 infections are only reported from humans, genotypes 3 and 4 can also infect animals (pigs, rabbits, wild boar, deer) and are considered zoonotic [4]. HEV causes an estimated 20 million human infections globally each year. Infections are typically acute and self-limiting; however, outcomes can be more severe and long-lasting in immunocompromised patients or during pregnancy. For example, case fatality rates of 20–25% have been observed in pregnant patients infected with HEV genotypes 1 or 2 [5]. The main route of transmission is faecal-oral. In developing countries human infections normally occur through contaminated water, while in developed countries the main route of transmission is via consumption of undercooked meat (usually pork) [6]. However, the identification of rabbit HEV (HEV-3ra) in immunocompromised patients in France without any link to the consumption of rabbit meat suggests possible alternative routes of transmission that have not yet been identified [7,8].

In 2020, HEV-3ra was detected for the first time in Australian domestic rabbits through RNA sequencing of liver samples [9]. Currently, little is known about the prevalence and distribution of HEV in domestic and wild rabbits in Australia. Due to the high number of wild rabbits in Australia and the increasing popularity of rabbits as pets, rabbit-associated HEV may pose a potential public health risk to the Australian population. In this study, we aimed to further understand the distribution and genetic variability of HEV in Australian rabbits to shed further light on the potential health risk at this human–animal interface.

## 2. Results

### 2.1. RT-qPCR Results and Seroepidemiology Show That Hepatitis E Virus is Widely Distributed in Australian Rabbit Populations

**Figure 1:**
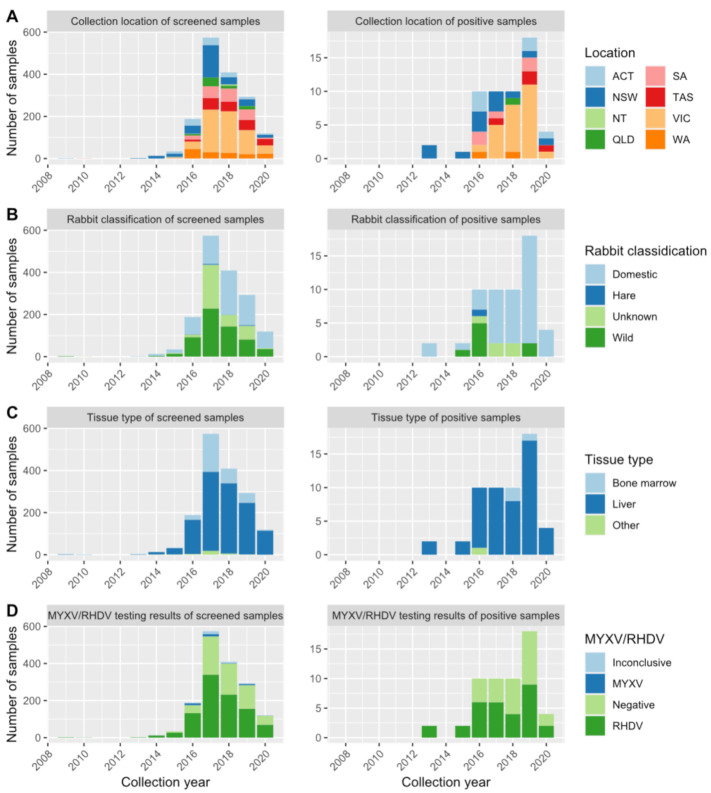
Metadata of samples screened for HEV via RT-qPCR (left) compared to those found positive via RT-qPCR (right). Samples were categorised by state (**A**), rabbit use (**B**), tissue type (**C**), and results of previous pathogen testing (**D**). MYXV myxoma virus; RHDV rabbit haemorrhagic disease virus (includes genotype GI.1 and GI.2 lagoviruses). Note that the y-axis scales differ between the left and right panels. WA—Western Australia, NT—Northern Territory, SA—South Australia, QLD—Queensland, NSW—New South Wales, VIC—Victoria, TAS—Tasmania, ACT—Australian Capital Territory.

To investigate the presence of HEV in Australian rabbits, 1635 RNA samples extracted from Australian wild and domestic rabbit liver or bone marrow samples were screened for HEV via RT-qPCR. Samples were collected previously for lagovirus surveillance and were first screened for rabbit haemorrhagic disease virus (RHDV) and myxoma virus (MYXV). Sample collection date, location, tissue, rabbit use, and the results of MYXV and RHDV testing of screened samples are summarized in Figure 1. Of the 1635 samples, 56 (3.4%) were positive for HEV RNA, with Ct (cycle threshold) values between 19.77 and 41.74. Positive samples were collected between December 2013 and May 2020 and were from all Australian states and territories except the Northern Territory (Figure 1). Overall, 42 of the 56 positive samples (75%) were from domestic rabbits, while eight samples (14.3%) were from wild rabbits, five samples (8.9%) were of unknown origin (wild or domestic) and one sample (1.8%) was from a hare. In 31 of 56 samples (55%) coinfection with RHDV was observed. This number is expected (χ^2^ = 0.27) due to the high number of RHDV positive samples in the dataset (60%); the HEV positivity was not significantly different between RHDV-positive vs. RHDV-negative rabbits (*p* = 0.6).

**Figure 2:**
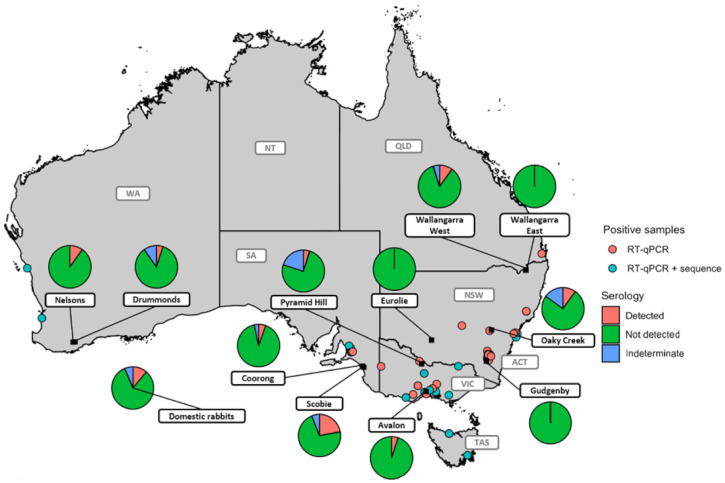
Map of HEV-positive tissue samples based on the RT-qPCR assay and sampling sites for HEV serology. Dots refer to samples from dead rabbits that tested positive in the RT-qPCR assay and are coloured by whether a near-complete genome sequence was obtained. Pie charts for each sampling site (black squares) display the number of detected, not detected and indeterminate samples. A summary of sample numbers and serology results for each monitoring site can be found in Table 1. WA—Western Australia, NT—Northern Territory, SA—South Australia, QLD—Queensland, NSW—New South Wales, VIC—Victoria, TAS—Tasmania, ACT—Australian Capital Territory.

To investigate the seroprevalence in wild and domestic rabbit populations we tested 442 serum samples (362 from wild rabbits and 80 from domestic rabbits) for the presence of HEV antibodies using a newly developed Luminex assay. Since true negative sera were not available to validate this assay, we used a conservative positivity cut-off (described in Materials and Methods). HEV antibodies were detected in 9/80 domestic rabbit samples (11.25%) and a further 6 (7.5%) returned an indeterminate result (Figure 2). For wild rabbits, a total of 362 serum samples from 11 different locations were tested, with HEV antibodies detected in 33 (9.11%) and a further 18 (4.97%) classified as indeterminate (Figure 2). Notably, HEV seroprevalence in wild rabbit populations varied widely between sites. For example, while HEV antibodies were not detected from Wallangarra East (Queensland), Eurolie (New South Wales), or Gudgenby (Australian Capital Territory), almost a quarter of samples from Scobie (South Australia) (22%) tested positive. Interestingly, while HEV antibodies were not detected at Wallangarra East, there were two positive detections and one indeterminate sample at Wallangarra West, only 2.5 km away. Furthermore, sera from two sites, Scobie and Coorong, were tested across four sampling times over three years (January, April, July, and October). The HEV seropositivity was highest at the summer/autumn sampling points (January and April) but this was not significantly different from spring or winter sampling timepoints. Results for each sample site are summarized in Table 1.

### 2.2. Australian HEV Ssequence Analysis Shows High Genetic Diversity with Minimal Phylogeographic Structure

**Figure 3:**
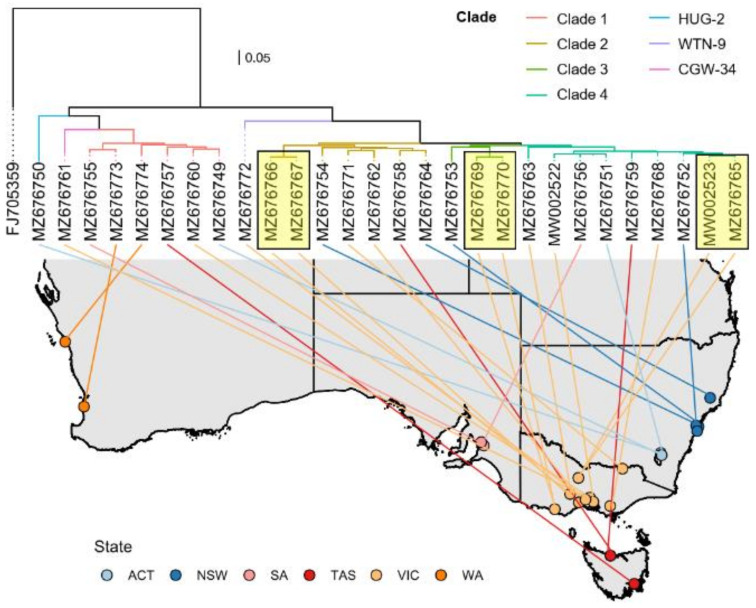
Phylogeographic analysis of Australian rabbit HEV sequences. A maximum likelihood phylogenetic tree was estimated using the best-fitted model (TIM2+F+I+G4) in IQ-TREE (v 1.6.12) [12] with 1000 ultra-fast bootstrap replicates. The tree was rooted along the branch leading to FJ05359 (HEV genotype 3 from a wild boar in Germany [13]). Colours of tree branches refer to the clades representing the 7 separate PrimalScheme assays. Lines connect the sampling location on the map to the position within the phylogenetic tree. Colours correspond to the states where samples were collected. Yellow rectangles show samples from rabbits that were co-housed. The scalebar represents substitutions per site. WA—Western Australia, SA—South Australia, QLD—Queensland, NSW—New South Wales, VIC—Victoria, TAS—Tasmania, ACT—Australian Capital Territory.

**Figure 4:**
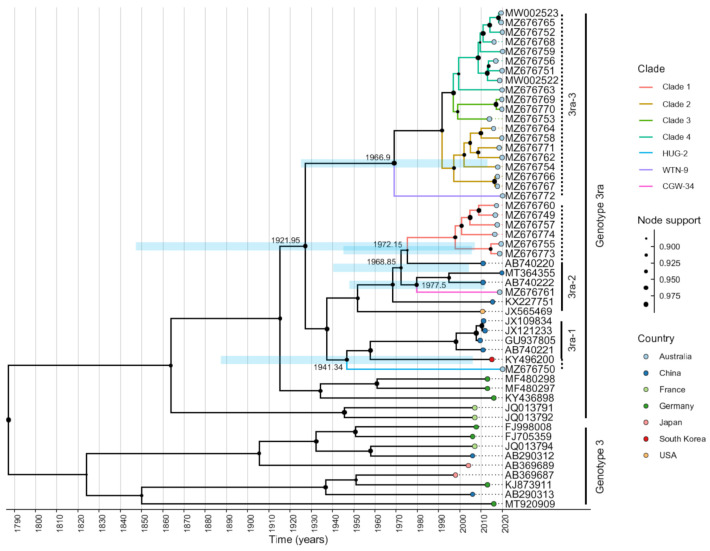
Time-resolved phylogenetic analysis of Australian HEV-3ra, global HEV-3ra and representative global whole-genome sequences of genotype 3 HEV. Genotype 3 reference sequences were based on those used by Smith et al. [14]. Tips are coloured according to the country of origin and node support is indicated by the size of the filled circles at internal nodes. Branches that lead to Australian sequences are coloured based on the PrimalScheme assay used for amplification. Blue horizontal bars correspond to the 95% highest posterior density (HPD), with the median indicated at the relevant node. The x-axis is given in years. Clade labels indicate genotype 3 non-rabbit HEV sequences and the rabbit-specific HEV-3ra sequences, including the previously defined subgenotypes 1 and 2 [15]. Dotted line clade labels show the extension of existing subgenotypes and newly defined one.

Based on HEV RT-qPCR Ct values, rabbit use, and tissue type, 48 samples were selected for RNA sequencing (‘metatranscriptomics’) to attempt to recover complete HEV genomes [9]. The number of HEV reads per sample ranged from 1 to 7597 reads (Table S1). Only 20 samples generated HEV read counts above 100, and for these samples an HEV genome coverage of >82% was reached. In 11 cases, a genome coverage of >99% was achieved. These sequences were used to generate seven overlapping tiled amplicon sequencing (PrimalScheme) assays with improved sensitivity compared to total RNA sequencing approaches [10] (Table S2). With this amplicon sequencing strategy, we were able to recover 28 near-complete HEV-ra genomes (complete coding sequence), two of which were already published in a previous study, and all of which were from domestic rabbit liver samples [9]. Based on sequence identity (and therefore the use of different PrimalScheme assays), Australian HEV-3ra sequences were distinguished into four clades (clade 1–4) plus three genomic singletons (HUG-2, WTN-9, CGW-34) that were genetically diverse and remained unclassified (Figure 3). No clear geographic structure was observed in the Australian HEV-ra phylogeny, however, samples submitted concurrently from rabbits that were co-housed clustered together with very high sequence identity (≥98.9%) (Figure 3). All Australian HEV sequences clustered with previously reported rabbit HEV sequences (Figure 4) and had a 93-nt insertion within the X domain of ORF1 that is characteristic of rabbit HEV sequences globally [11]. Nucleotide sequence identity between Australian HEV-3ra sequences ranged from 82.7 to 99.6%, while for international HEV-3ra sequences, this value ranged from 79.5 to 91%. Based on publicly available sequences, the closest international HEV-ra sequences originated from China and South Korea (Figure 4).

### 2.3. Time-Structured Phylogenetic Analysis Indicates Multiple Independent Introductions of HEV into Australia

A time-resolved phylogenetic analysis was conducted to infer the possible numbers of introductions of HEV-3ra into Australia and the timing of introduction events. While clades 2–4 and WTN-9 span considerable genetic diversity (88.56–99.56% nucleotide identity), no globally available HEV-3ra sequences fall within this branch of Australian HEV-3ra and these sequences could be considered as subgenotype (sg) 3, according to the system proposed by Nicot et al. [15]. Clade 1 and CGW-34 formed a monophyletic cluster with Chinese and US HEV-3ra sg 2 sequences, with CGW-34 being distinct from Clade 1 sequences. This sg now spans 87.88–98.55% nucleotide identity. HUG-2 clustered most closely with HEV-3ra sg 1 sequences from China and South Korea, differing from other sequences in this sg by up to 13.74% at the nucleotide level. Inter-sg nucleotide identity ranged from 84.34 to 88.70% between sg 1 and 2, 82.99 to 85.49% between sg 1 and 3, and 82.69 to 87.57% between sg 2 and 3. These findings together suggest that HEV has been introduced into Australia on several independent occasions (Figure 4). At least two introductions date back to the late 1970s (95% highest posterior density (HPD) 1953–1991 and 1958–1992). It is possible that an unsampled lineage associated with CGW-34 may have resulted from yet another introduction event in the 1980s (95% HPD 1962–1994). The sequence HUG-2 is clearly independent and forms a distinct branch that is highly divergent from all other Australian HEV sequences. This introduction also dates back further than the other introductions, to the 1950s (95% HPD 1923–1979). The timeframes in this analysis should, however, be interpreted cautiously because of the overlapping HPDs. In addition, HEV-3ra isolates are extremely undersampled globally, and the scarcity of available sequences adds further uncertainty to these numbers.

In addition to time-structured analysis, phylogenetic trees of ORF1, ORF2 and whole genome sequences were assessed for evidence of recombination events. No major incongruencies were detected between the ORF1, ORF2 and whole genome phylogenies, confirming that recombination (at least at the ORF level) was not a feature of these genomes (Figure S1). An evolutionary rate of 3.3 × 10^−3^ substitutions per site per year was estimated (Figure S2), similar to previously reported evolutionary rate estimates for HEV [16].

## 3. Discussion

In this study, we screened Australian wild and domestic rabbits for evidence of exposure to HEV. We found a 3.4% positivity based on RT-qPCR screening of tissue samples. This sets a lower bound to the true positivity, since the sensitivity of any diagnostic test will always be <100% and liver RNA extracts particularly are known to be prone to PCR inhibition. From the positive samples we recovered 28 near-complete genomes using a combination of RNA sequencing and amplicon-based sequencing strategies. Using a newly established HEV Luminex serological assay, we found that seroprevalence varied from 0% to 22%, depending on the source population. Taken together, these findings suggest that HEV-3ra infection is relatively common in both wild and domestic rabbit populations throughout Australia. Although one hare sample tested positive by RT-qPCR, no genome could be amplified from this sample. Additional hare samples are required to determine if HEV circulates endemically within the Australian hare populations. Most detections of HEV were in Victoria, however this is most likely due to sampling bias, as Victoria has large domestic and wild rabbit populations and most samples were collected in Victoria. 

The HEV positivity rate (by RT-qPCR) varied considerably by year (1.74–6.14%). Although fewer samples were submitted for 2019 (293 samples) compared to previous years (409 and 574 samples, respectively), detections of HEV in 2019 (6.14%) exceeded those for 2017 (1.74%) and 2018 (2.44%). This might indicate that HEV-3ra infections are increasing over time, but further monitoring in future years is needed to confirm this trend. We identified a high incidence of RHDV and HEV coinfections (31/56), however, noting that 60% of all rabbit samples in this study tested positive for RHDV, a 55% proportion of RHDV infections in HEV positive samples is expected and therefore likely to be a coincidental finding rather than an indication of a biological interaction. Statistical analysis does not support a significantly different risk of HEV infection in RHDV-positive samples. However, the biological relevance of HEV and RHDV coinfection may warrant further investigation considering the importance of RHDV as biocontrol in Australia. 

Another sampling bias was the predominance of samples from domestic rabbits, with only 25% of liver samples being collected from wild rabbits. Veterinarians and pet owners monitor domestic animals closely and are more likely to submit samples in the case of disease or death, while wild rabbits are mostly collected opportunistically [17,18,19]. Furthermore, sample quality was better from domestic rabbits compared to wild rabbits, because typically samples from domestic rabbits were collected fresh while wild rabbit samples were often degraded following environmental exposure before collection. This was demonstrated by comparing RT-qPCR Ct values, which were higher in wild (75% of samples had a Ct value > 35) than in domestic rabbits (13% of samples had a Ct value >35). However, this difference was not statistically significant (*p* = 0.14), likely because only 4 wild rabbit samples were positive in this study. Sample quality, especially from wild rabbits, frequently did not meet the quality standards required for efficient genome amplification. The high genetic diversity observed in HEV sequences is likely also an important factor limiting the recovery of full genomes, with current HEV-3ra genome sequences from Australian rabbits ranging from 82.7 to 99.6% sequence identity. Correspondingly, only 28 of 56 positive samples could be amplified with the primer sets inferred by RNA sequencing. 

Based on the results of the time-resolved phylogenetic analysis, HEV was likely introduced at least three times into Australia. Notably, based on the relative scarcity of HEV whole genome sequences globally and the high genetic diversity of Australian sequences, it is likely that the number of introductions may indeed be higher. The available sequences in GenBank indicate that the HEV introductions into Australia most likely originated in China. However, the number of sequences from China far exceed those available from other countries, and therefore introductions of HEV into Australia from other countries cannot be excluded. Furthermore, the Australian HEV-3ra genome sequences showed no clear geographic clustering. For example, the three sequences obtained from the Australian Capital Territory were highly divergent, falling within Clade 1, Clade 4, and as a genomic singleton, respectively. This suggests that there are no obvious barriers between different rabbit populations, at least in the domestic rabbits from which sequences were obtained. In animal populations with distinct spatial barriers, this can be observed in the phylogeographic structure. For example, in a study of feline immunodeficiency virus in Californian bobcats, in populations whose movement was restricted due to highways, a clear geographic pattern could be observed in the phylogeny, while populations without those spatial barriers showed no such structure [20]. Noting that all sequences were derived from domestic rabbit samples, this could suggest a high rate of transportation of domestic rabbits across the continent, either for meat production, showing, breeding, or as pets. However, an extended study of HEV-3ra in wild rabbits in Australia would be necessary in the future to investigate if the lack of transportation as outlined above supports a phylogeographic structure. Undersampling of HEV in Australian rabbits, nationally and internationally, also contributes to the lack of a phylogeographic structure. Finally, although recombination events of rabbit HEV have been reported in the past [21], this was not observed in current HEV-3ra sequences (Figure S1). 

Despite the zoonotic potential of HEV and the abundance of rabbits in Australia, to date no HEV infections in humans with HEV-3ra clade strains have been reported in this country. Although approximately 30–60 human cases of HEV infection are detected each year in Australia [22], the majority are linked to international travel. Only one published case of a locally acquired cluster in 2013/2014, due to the consumption of contaminated pork meat, has been reported [23]. While rabbits are farmed for meat production in Australia, the market share is very small, estimated at 157 tonnes dressed weight (~131,000 rabbits) in 2003 [24]. A recent study of HEV cases identified in humans in Australia between 2016–2019 determined that approximately 63% cases were genotype 1, a strain which is exclusively found in humans, 33.3% were genotype 3 and 3% were genotype 4. None of the 22 genotype 3 isolates (the same genotype as HEV-3ra) were associated with the rabbit strain of HEV [22]. Although the risk to humans from HEV through transmission from rabbits seems to be low, the rabbit/human interface is quite large and further surveillance may be warranted, including the testing of fecal samples, to better assess this potential risk. This zoonotic risk may be enhanced with the potential identification of recombinants or new variants of HEV-3ra in the future. Notably, a recent publication from Germany discovered an HEV strain isolated from rabbits that lacked the characteristic 93-nt insertion in the X domain of ORF1, thus spanning the interface between human and rabbit strains according to phylogenetic analyses [25]. This may suggest the emergence of new HEV strains that could pose an increased risk to the human population. Therefore, further surveillance would clearly be beneficial to increase our understanding of the number and timing, as well as the source, of HEV-3ra introductions into Australia and to rapidly detect new emerging viruses.

## 4. Materials and Methods

### 4.1. Sample Collection

Sample collection for lagovirus surveillance was described previously [17]. No animal ethics approvals are required for sampling rabbits that are found dead in Australia. Samples were provided either fresh frozen or stored in an RNA stabilization solution [26,27]. RNA from tissue samples (liver and bone marrow) was extracted using the Maxwell® RSC instrument (Promega) in combination with the Maxwell® RSC SimplyRNA Tissue Kit (Promega) according to the manufacturer’s instructions. Prior to extraction, tissue was homogenised using glass beads and a Precellys 24-dual tissue homogenizer (Bertin Technologies).

### 4.2. RT-qPCR

An HEV-specific RT-qPCR was used to screen RNAs for the presence of HEV RNA [28]. The Luna^®^ Universal Probe One-Step RT-qPCR Kit (New England Biolabs) was used according to the manufacturer’s instructions. In short, a 10 µL reaction contained 1x Luna Universal Probe reaction mix, 1x RT enzyme mix, a final concentration of 0.4 µM for each primer (HEV-F: 5′-GGTGGTTTCTGGGGTGAC-3′, HEV-R: 5′-AGGGGTTGGTTGGATGAA-3′) and 0.2 µM for the probe (5′-FAM-TGATTCTCAGCCCTTCGC-BHQ-3′) with the addition of 1 µL of extracted RNA. Cycling conditions were 55 °C for 10 min, 95 °C for 2 min, followed by 45 cycles of 95 °C for 15 s, 55 °C for 30 s and 72 °C for 15 s. In vitro RNA transcripts covering the target region (Table S3) were used as a positive control. A no template control served as a negative control.

### 4.3. Sequencing

Forty-eight positive samples were prepared for RNA sequencing using the NEB-Next^®^ Ultra™ II RNA Library Prep Kit for Illumina^®^ (New England Biolabs, Ipswich, MA, USA) including an rRNA depletion step (NEBNext® rRNA Depletion Kit (Human/Mouse/Rat), New England Biolabs). Sequencing was performed on an Illumina NovaSeq6000 instrument (SP300 cycle flow cell) at the Biomolecular Resource Facility (BRF), The John Curtin School of Medical Research, Australian National University.

Based on sequences acquired through RNA sequencing, seven sets of tiled amplicon primers [10] were designed (Table S2). HEV was amplified from RNA using the One-step Ahead RT-PCR Kit (Qiagen). Amplicons were pooled and purified with magnetic beads (Bioline) and libraries were constructed using the NEBNext^®^ Ultra™ II FS DNA Library Prep Kit for Illumina^®^ (New England Biolabs). Libraries were sequenced on an Illumina MiSeq instrument (300 cycles, v2). Gaps were closed with primer combinations spanning the unsequenced regions (Table S4) using the One-step Ahead RT-PCR Kit (Qiagen) following the manufacturer’s instructions. Purified amplicons were sequenced in both directions by Sanger sequencing at the BRF to generate a consensus sequence that was merged with the partial amplicon sequence. 

### 4.4. Sequence Data Analysis

The quality of the RNA-Seq and amplicon sequencing data was checked using FastQC (v0.11.08). Low quality reads (SLIDINGWINDOW:4:32, MINLEN:50) were removed, ends were trimmed (HEADCROP:15) using Trimmomatic (v0.38) [29] and paired reads were merged with FLASh (v1.2.11) [30]. Cleaned sequence reads from the metatranscriptomic data were then mapped against the rabbit genome (GCA_000003625.1 OryCun2.0) using Bowtie2 (v2.2.9) [31] to exclude host-related reads from further analysis. The remaining reads were subsequently mapped to a previously sequenced HEV genome (MW002523) with Bowtie2 (v2.3.0), as implemented in Geneious Prime (v2020.2.2). For amplicon sequencing data, cleaned reads were mapped directly to the HEV reference (MW002523). Sanger sequences were trimmed based on quality, and forward and reverse sequences were aligned. The resulting consensus sequence was aligned to the respective HEV sequence. All sequences that were used for further analysis within this study were deposited in GenBank (Accession numbers MZ676749–MZ676774).

### 4.5. Phylogeographic Analysis

Australian HEV sequences and an HEV genotype 3 sequence from a wild boar in Germany (FJ705359) [13] were aligned using MAFFT (v7.450) [32] as implemented in Geneious Prime (v2020.2.2). Sequences were trimmed by removing the 3′- and 5′-UTRs. A maximum likelihood phylogenetic tree was estimated using the best-fitted model (TIM2+F+I+G4) as determined in IQ-TREE (v1.6.12) [12] with 1000 ultra-fast bootstrap replicates. The tree was rooted along the branch leading to FJ705359. 

### 4.6. Bayesian Evolutionary Analyses Analysis

Australian HEV sequences, complete rabbit HEV-3ra sequences available in Genbank, and reference sequences for other HEV genotypes according to Smith et. al [14] were aligned using MAFFT (v7.450) [32] as implemented in Geneious Prime (v2020.2.2). Sequences were trimmed by removing the 3′- and 5′-UTRs. Sequences were removed if no temporal data were available. Alignments of whole HEV genome sequences, ORF1 and ORF2 were screened using TempEst [33] to confirm sufficient temporal signal as follows. Maximum likelihood phylogenies for ORF1, ORF2 and whole genome sequences were estimated using the best-fitted model as determined in IQ-Tree (v1.6.12) with 1000 ultra-fast bootstrap replicates. These were used as input to construct linear regressions of root-to-tip distances against sampling time. Furthermore, phylogenetic trees for all three alignments were compared to check for evidence of recombination (Figure S1). The strongest temporal signal (correlation coefficient = 0.56) was found for the whole HEV genome alignment, which was used for all further analyses. A Bayesian Markov chain Monte Carlo (MCMC) approach was used to infer a time-scaled phylogeny. Marginal likelihood estimations (MLE), as implemented in BEAUti (v1.10.4), were used to assess the most appropriate clock prior (strict versus uncorrelated log-normally distributed (UCLD)) and tree prior (Gaussian Markov random field Bayesian skyride model versus constant size coalescent versus exponential coalescent). A substitution model (GTR+F+I+G4), as inferred by using the Modeltest [34] as implemented in IQ-Tree (v1.6.12) [12], was used for each MLE. Best MLE values were reached with a UCLD clock prior and the Bayesian skyride model and were used for a subsequent Bayesian Evolutionary Analysis Sampling Tree (BEAST) (v1.10.4) [35]. The analysis was run twice to convergence (ESS > 200) to confirm consistency. 

### 4.7. Serology

Sera at a dilution of 1:50 in PBS was added to magnetic microsphere beads (MagPlex, BioRad) coupled with Hepatitis E recombinant capsid protein (yeast-expressed, Cusabio) and incubated with shaking at room temperature (RT) for 30 min in a 96-well plate. Bound antibody was detected following the addition of biotinylated Protein A together with biotinylated Protein G (ThermoFisher), followed by the addition of streptavidin-phycoeryrthin (ThermoFisher). Washing of the beads in PBS-T (phosphate-buffered saline + 0.05% Tween 20) (three times) using a magnetic plate washer (Tecan) was performed prior to the addition of reagents at each step, with all incubations performed in a volume of 100 µL, at RT with shaking for at least 30 min in the dark. The median fluorescent intensity (MFI) was read on a MagPix machine (Luminex). Based on cut-off values, samples were categorized as HEV antibodies detected, not detected or indeterminate. Forty-five serum samples from a domestic rabbit breeding colony were selected as negative samples to calculate a cut-off value. Three times the mean MFI was used as a preliminary cut-off for negative samples. Double the cut-off value was used to account for uncertainty, since no verified HEV-negative samples were available for testing. Samples within this range between 3 and 6 times the mean MFI of presumed negative sera were called inconclusive. A commercial HEV positive serum (IDvet), diluted 1:10 in PBS, was used as a positive control. Wild rabbit serum samples from Coorong (92 samples) and Scobie (86 samples) were first selected from 4 sampling times throughout each year (January, April, July, and October) from 2016–2018. Summer/autumn sampling (January and April) were found to have the highest HEV seropositivity (although this was not significantly different to winter/spring sampling), therefore 20 samples from an additional 9 sites collected in January 2017 and 2018 were subsequently selected for screening. All wild rabbit serum samples were collected during a previous monitoring program [36]. In addition, 80 residual serum samples from domestic rabbits were obtained from ComPath at SAHMRI (South Australia Health & Medical Research Institute).

### 4.8. Visualisation and Atatistical Analysis

All figures were plotted in R (v4.1.0) [37] using packages ggplot2 (v3.3.5) [38], ggnewscale (v0.4.5) [39], scatterpie (v0.1.6) [40], raster (v3.4-13) [41], treeio (v1.16.1) [42], ggtree (v3.0.2) [43], phytools (v0.7-80) [44], tidyverse (v1.3.1) [45], cowplot (v1.1.1) [46], RColorBrewer (v1.1-2) [47], scales (v1.1.1) [48] and data.table (v1.14.0) [49].

A Pearson’s Chi-squared test with Yates’ continuity correction was calculated in R (v4.1.0) to determine if co-infections of HEV-3ra and RHDV were significantly different from expected values within the dataset.

## Figures and Tables

**Table 1:** Summary of serology results.

Site	State *	Detected % (Number of Samples)	Not Detected % (Number of Samples)	Indeterminate % (Number of Samples)
Oaky Creek	NSW	10 (2/20)	75 (15/20)	15 (3/20)
Coorong	SA	5.43 (5/92)	91.30 (84/92)	3.26 (3/92)
Scobie	SA	22.09 (25/86)	72.09 (68/86)	5.81 (11/86)
Wallangarra East	QLD	0 (0/24)	100 (24/24)	0 (0/24)
Avalon	VIC	5 (1/20)	95 (19/20)	0 (0/20)
Pyramid Hill	VIC	5 (1/20)	75 (15/20)	20 (4/20)
Drummonds	WA	5 (1/20)	85 (17/20)	10 (2/20)
Nelsons	WA	10 (2/20)	90 (18/20)	0 (0/20)
Eurolie	NSW	0 (0/20)	100 (20/20)	0 (0/20)
Gudgenby	ACT	0 (0/20)	100 (20/20)	0 (0/20)
Wallangarra West	QLD	10 (2/20)	85 (17/20)	5 (1/20)
Total (wild rabbits)		9.11 (33/362)	85.91 (311/362)	4.97 (18/362)
domestic rabbits		11.25 (9/80)	81.25 (65/80)	7.5 (6/80)

* WA—Western Australia, SA—South Australia, QLD—Queensland, NSW—New South Wales, VIC—Victoria, ACT—Australian Capital Territory.

## Data Availability

All sequences that were generated in this study and used for further analysis within this study were deposited in GenBank (Accession numbers MZ676749–MZ676774).

## Supplementary Materials

The following are available online at https://www.mdpi.com/article/10.3390/pathogens10121637/s1, Figure S1: Linear regression of the root-to-tip distance of whole-genome HEV sequences against sampling time used for subsequent BEAST analyses; Figure S2: Comparison of phylogenetic trees of ORF1 and ORF2 of HEV. Trees were inferred by using the best fitted model (GTR+F+R3) in IQ-Tree; Table S1: Summary of sequenced samples; Table S2: Primers used for Primalscheme assays; Table S3: Sequence of positive control for RT-qPCR assay; Table S4: Primers used for subsequent Sanger sequencing.
