# Supplementary material for: Distribution and Genetic Diversity of Hepatitis E Virus in Wild and Domestic Rabbits in Australia
Source: Pathogens. 2021 Dec 17;10(12):1637. doi: 10.3390/pathogens10121637 (PMC8709427; doi:10.3390/pathogens10121637)
Supplement: Supplementary file 1 [file pathogens-10-01637-s001.zip › pathogens-1497175-supplementary.pdf]

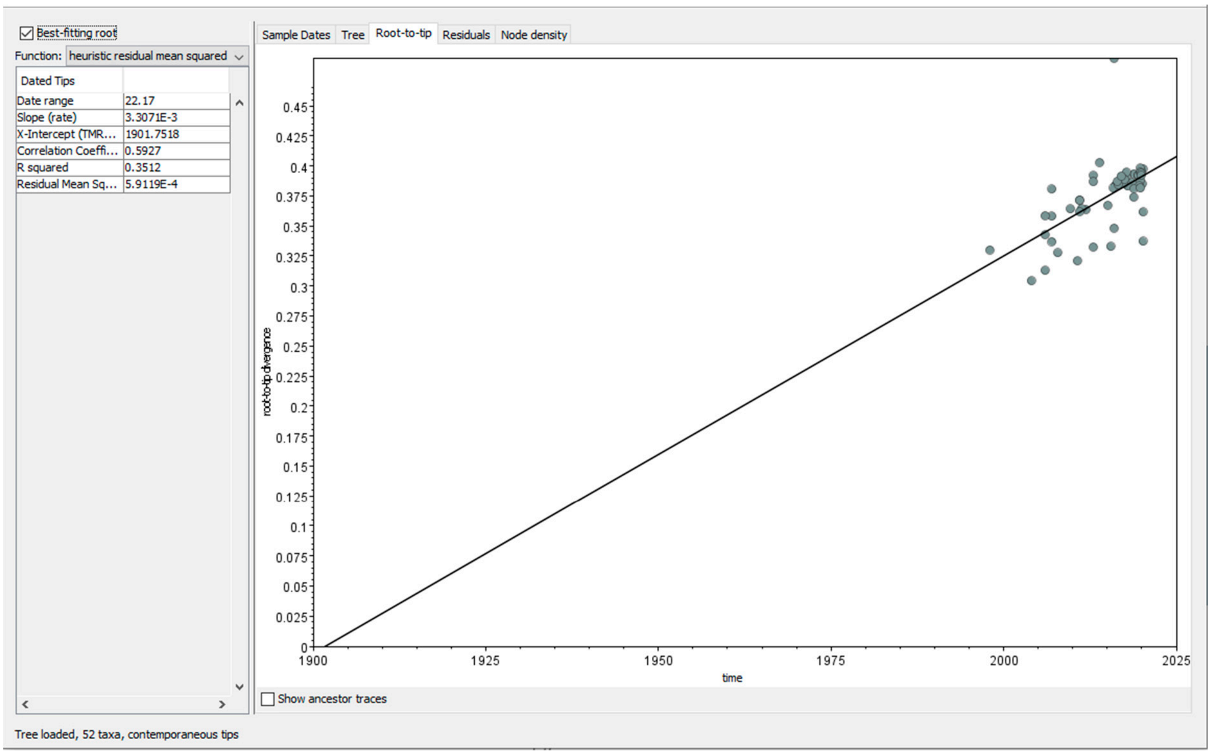

Supplement Figure S1: Linear regression of the root-to-tip distance of whole-genome HEV sequences against sampling time used for subsequent BEAST analyses.

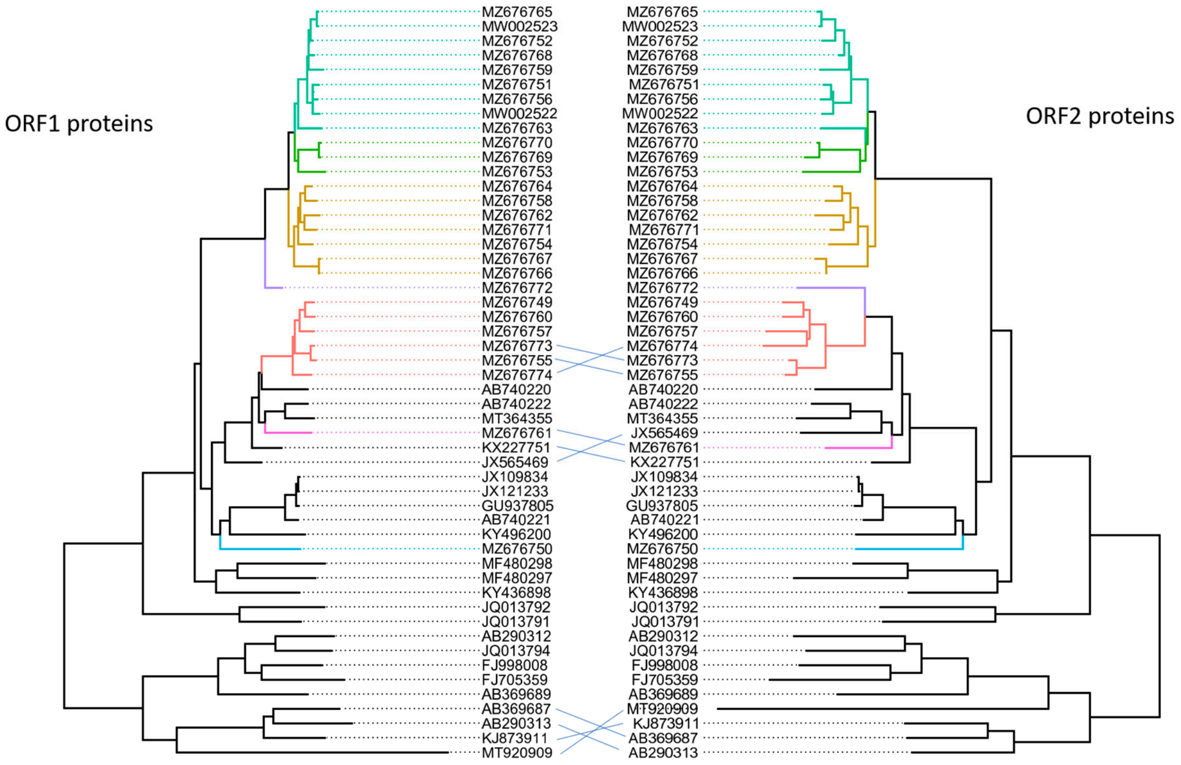

Supplement Figure S2: Comparison of phylogenetic trees of ORF1 and ORF2 of HEV. Trees were inferred by using the best fitted model (GTR+F+R3) in IQ-Tree.
